# Association of Early Pregnancy Inflammatory Indices with Preterm Birth and Perinatal Outcomes in Pregnancies with Pregestational Diabetes

**DOI:** 10.3390/jcm14196834

**Published:** 2025-09-26

**Authors:** Serenat Eris Yalcin, Mustafa Rasit Ozler, Yakup Yalcin, Erkan Saglam, Ebubekir Sıddık Yilmaz, Nuray Nerez

**Affiliations:** 1 Department of Obstetrics and Gynecology, Faculty of Medicine, Uludağ University, 16285 Bursa, Turkey; yakupyalcin@uludag.edu.tr; 2 Department of Obstetrics and Gynecology, Bursa City Hospital, 16900 Bursa, Turkey; m.rasitozler@gmail.com (M.R.O.); dresaglam@gmail.com (E.S.); drebubekiryilmaz71@gmail.com (E.S.Y.); nereznuray@gmail.com (N.N.)

**Keywords:** pregestational diabetes mellitus, inflammatory indices, neutrophil-to-lymphocyte ratio, platelet-to-lymphocyte ratio, systemic immune–inflammation index, preterm birth, perinatal outcomes, biomarkers, first trimester, risk stratification

## Abstract

**Background/Objectives:** We aimed to investigate the relationship between early pregnancy inflammatory indices and adverse perinatal outcomes in pregnancies complicated by pregestational diabetes mellitus (PREGDM) and to evaluate their predictive value, particularly for preterm birth and composite adverse perinatal outcome (CAPO). **Methods:** This retrospective study included 140 women with PREGDM and 140 age-matched controls. Early pregnancy (8–14 weeks) inflammatory indices [the neutrophil-to-lymphocyte ratio (NLR), platelet-to-lymphocyte ratio (PLR), monocyte-to-lymphocyte ratio (MLR), systemic immune–inflammation index (SII), systemic inflammation response index (SIRI)], and C-reactive protein (CRP) were calculated from complete blood counts. Associations with preterm birth and CAPO were analyzed using correlation, ROC analysis, and multivariate logistic regression adjusted for maternal age, BMI, HbA1c, and parity. **Results:** All inflammatory indices were significantly higher in the PREGDM group compared to controls (*p* < 0.05). Preterm birth, macrosomia, cesarean delivery, NICU admission, and CAPO were more frequent in PREGDM pregnancies (all *p* < 0.001). On univariate analysis, NLR, PLR, MLR, SII, and SIRI were significantly higher in women with preterm birth (*p* < 0.05), but not in those with CAPO. ROC curves showed modest discriminative ability of NLR, PLR, and SII for preterm birth (AUC 0.64–0.66), while AUCs for CAPO prediction were close to 0.5. In multivariate analysis, inflammatory indices were not independent predictors of either outcome. Only HbA1c (OR: 1.71, 95% CI 1.20–2.43, *p* = 0.003) and parity (OR: 1.62, 95% CI 1.08–2.45, *p* = 0.021) independently predicted preterm birth, and similarly HbA1c (OR: 1.68, 95% CI 1.14–2.46, *p* = 0.008) and parity (OR: 1.49, 95% CI 1.02–2.15, *p* = 0.037) predicted CAPO. **Conclusions:** Early pregnancy inflammatory indices were associated with preterm birth in univariate analyses but lost significance after adjustment for maternal and metabolic risk factors. HbA1c and parity remained the only independent predictors of adverse outcomes in PREGDM pregnancies. Inflammatory indices may provide supplementary information but should not be used as stand-alone predictors; they may instead be incorporated into multiparametric models with established clinical and metabolic markers to improve risk stratification.

## 1. Introduction

Pregestational diabetes mellitus (PREGDM), defined as diabetes present before conception, significantly increases the risk of adverse maternal and perinatal outcomes. Women with PREGDM are more prone to develop hypertensive disorders, preterm labor, fetal growth abnormalities, stillbirth, and neonatal metabolic disturbances [1,2]. The pathogenesis of these complications is complex and multifactorial, involving hyperglycemia, placental dysfunction, oxidative stress, and systemic inflammation [3,4,5,6]. Long-standing metabolic imbalance in PREGDM contributes not only to endothelial dysfunction and impaired uteroplacental circulation but also to chronic immune activation, which may predispose to both maternal and fetal complications.

In recent years, increasing attention has been paid to the role of systemic inflammation in obstetric complications [7,8,9,10,11]. Chronic low-grade inflammation in diabetic patients may contribute to impaired placental perfusion, endothelial dysfunction, and immune–mediated mechanisms leading to preterm labor [3,4,5]. Moreover, systemic inflammation has been implicated in the pathophysiology of preeclampsia, fetal growth restriction, and gestational diabetes, suggesting that hematologic inflammatory indices could represent a common mechanistic link across multiple pregnancy complications. Consequently, identifying reliable and accessible markers of inflammation has become a major research focus, particularly in high-risk obstetric populations such as women with PREGDM.

Several hematologic indices derived from complete blood counts, including the neutrophil-to-lymphocyte ratio (NLR), platelet-to-lymphocyte ratio (PLR), monocyte-to-lymphocyte ratio (MLR), systemic immune–inflammation index (SII), and systemic inflammation response index (SIRI), have been identified as potential indicators of systemic inflammation. These indices have shown predictive value in various medical fields, including oncology, cardiology, and infectious diseases, and are now being increasingly investigated in obstetrics [12,13,14]. Their advantages include low cost, wide availability, and the ability to reflect real-time systemic immune activity, making them attractive candidates for integration into routine prenatal risk assessment.

Although several studies have examined these indices in relation to preeclampsia, fetal growth restriction, gestational diabetes, and preterm premature rupture of membranes, results have been inconsistent and predictive accuracy remains modest [11,12,13]. In pregnancies complicated by PREGDM, evidence is even more limited and mostly restricted to analyses performed in late gestation [14,15]. Importantly, early pregnancy remains underexplored, despite being a critical window for placental development and maternal immune adaptation. Since interventions initiated early in gestation may have the greatest potential to improve maternal and neonatal outcomes, clarifying whether early hematologic inflammatory indices can serve as early warning markers in PREGDM pregnancies is of considerable clinical relevance.

Therefore, the objectives of this study were to (1) investigate differences in inflammatory indices between PREGDM and healthy pregnancies, (2) evaluate the associations between early pregnancy (8–14 weeks) inflammatory indices and adverse perinatal outcomes, and (3) assess the predictive performance of these markers, particularly for preterm birth and composite adverse perinatal outcome (CAPO). By addressing these aims, we sought to clarify the clinical significance of hematologic inflammatory indices and their potential role in prenatal risk stratification.

## 2. Materials and Methods

### 2.1. Study Design and Participants

This retrospective observational study included pregnant women with pregestational diabetes mellitus (PREGDM) and a control group of normoglycemic women who delivered at a tertiary care facility between January 2022 and January 2025. The study was conducted in accordance with the Declaration of Helsinki and approved by the Ethics Committee of Bursa City Hospital (Approval No: 2025-7/2).

Inclusion criteria were singleton pregnancy, delivery after 24 weeks of gestation, and availability of first-trimester complete blood count, CRP values, and perinatal outcome data. Exclusion criteria were multiple pregnancy, chronic kidney disease, autoimmune disorders, cancer, prior systemic inflammatory conditions, or incomplete medical records.

The PREGDM group comprised women diagnosed with type 1 or type 2 diabetes before conception, confirmed Via medical records or pre-pregnancy laboratory documentation, according to American Diabetes Association (ADA) diagnostic criteria [16]:

Type 1 diabetes mellitus (T1DM): characterized by autoimmune β-cell destruction and absolute insulin deficiency. Diagnostic features included early age of onset, low or undetectable fasting C-peptide levels (<200 pmol/L), and positivity for pancreatic autoantibodies (e.g., GAD65, IA-2, ZnT8, or insulin autoantibodies), when available. The clinical course is typically acute, with a high likelihood of insulin dependence soon after diagnosis.

Type 2 diabetes mellitus (T2DM): defined as diabetes occurring in the absence of autoimmune markers, usually associated with overweight or obesity and insulin resistance. Patients generally present in adulthood, although diagnosis may occur at any age. Fasting C-peptide is usually preserved or elevated (>600 pmol/L). Diagnostic thresholds included one or more of the following (confirmed on repeat testing unless unequivocal hyperglycemia was present): HbA1c ≥ 6.5% (48 mmol/mol), fasting plasma glucose ≥126 mg/dL (7.0 mmol/L), 2-h plasma glucose ≥200 mg/dL (11.1 mmol/L) during a 75-g oral glucose tolerance test (OGTT), or random plasma glucose ≥200 mg/dL (11.1 mmol/L) with classic symptoms of hyperglycemia or hyperglycemic crisis.

The control group consisted of healthy pregnant women without diabetes or chronic conditions, matched 1:1 to the study group based on maternal age (±2 years). As this was a retrospective study, all eligible women meeting inclusion criteria during the study period were included. Post Hoc power analysis based on the observed difference in mean NLR between groups indicated that the achieved sample size (*n* = 140 per group) provided >80% power at α = 0.05.

### 2.2. Data Collection and Laboratory Measurements

Maternal demographic characteristics, obstetric history, and laboratory data were retrieved from electronic medical records. All blood samples were collected between 8 and 14 weeks of gestation during the first hospital visit. Complete blood count (CBC): measured using an automated hematology analyzer (Sysmex XN-1000, Sysmex Corp., Kobe, Japan). HbA1c: measured by high-performance liquid chromatography (HPLC) (Bio-Rad D-100, Hercules, CA, USA). CRP: determined by immunoturbidimetric assay (Roche Cobas c702, Basel, Switzerland).

Inflammatory indices were calculated as follows:

NLR = neutrophil count/lymphocyte count

PLR = platelet count/lymphocyte count

MLR = monocyte count/lymphocyte count

SII = (neutrophil × platelet)/lymphocyte

SIRI = (neutrophil × monocyte)/lymphocyte

### 2.3. Outcomes

Adverse perinatal outcomes included preterm delivery (<37 weeks), low Apgar scores (<7 at 1st or 5th min), neonatal intensive care unit (NICU) admission due to neonatal hypoglycemia, respiratory distress syndrome (RDS), or other complications, birthweight below the 10th percentile for gestational age, macrosomia (birthweight > 4000 g), and perinatal mortality (intrauterine or early neonatal death). A composite adverse perinatal outcome (CAPO) was defined as the occurrence of any of the aforementioned outcomes.

Demographic characteristics, inflammatory markers, and perinatal outcomes were compared between the PREGDM group and healthy controls. In the second part of the study, the PREGDM group was subdivided into outcome-based subgroups (Preterm Vs. Term delivery and CAPO-Positive Vs. CAPO-Negative), and clinical findings and inflammatory indices were compared.

### 2.4. Statistical Analysis

Analyses were performed using IBM SPSS Statistics v25.0 (IBM Corp., Armonk, NY, USA). Categorical variables were presented as numbers and percentages, while continuous variables were expressed as mean ± standard deviation (SD) or median (IQR), as appropriate. Independent *t*-tests or Mann–Whitney U tests were used for continuous variables, and chi-square or Fisher’s exact tests were used for categorical variables.

Associations between perinatal outcomes and inflammatory indices were initially assessed using Spearman correlation analysis. Receiver operating characteristic (ROC) curves were generated to evaluate the univariate discriminative performance of inflammatory indices (AUC values and optimal cut-off points). To determine independent predictive value, multivariable logistic regression analyses were performed, adjusting for potential confounders including maternal age, BMI, HbA1c, and parity. A *p*-value < 0.05 was considered statistically significant.

## 3. Results

This study included 280 pregnant women: 140 with PREGDM and 140 healthy controls. Among the 140 women with pregestational diabetes, 63 (45.0%) had type 1 diabetes mellitus and 77 (55.0%) had type 2 diabetes mellitus. Women in the PREGDM group had significantly higher BMI, gravida, parity, abortus, HbA1c, and fasting blood glucose levels compared to controls (all *p* < 0.0001).

All inflammatory indices (NLR, PLR, MLR, SII, SIRI, and CRP) were significantly higher in the PREGDM group compared to controls, indicating a heightened systemic inflammatory state (*p* < 0.05) (Table 1).

Preterm birth (30% Vs. 5.7%, *p* < 0.0001), macrosomia (30% Vs. 4.3%, *p* < 0.0001), cesarean delivery (74.3% Vs. 44.3%, *p* < 0.0001), NICU admission (21.4% Vs. 8.6%, *p* = 0.004), and CAPO (62.9% Vs. 18.6%, *p* < 0.0001) were all significantly higher in the PREGDM group. PREGDM pregnancies also had significantly lower Apgar scores at the 1st and 5th minutes (*p* < 0.0001) (Table 1).

When stratified by CAPO presence, no significant differences were observed in inflammatory marker levels between CAPO-positive and CAPO-negative patients. Median NLR values were similar (3.92 Vs. 3.57; *p* = 0.2705), as were median PLR (139.08 Vs. 138.15; *p* = 0.5532), MLR (0.31 Vs. 0.32; *p* = 0.5303), SII (868.33 Vs. 885.05; *p* = 0.5474), and SIRI (1.86 Vs. 1.85; *p* = 0.5360). Median CRP levels were higher in CAPO-positive cases (2.58 Vs. 2.01), but this difference did not reach statistical significance (*p* = 0.0818). These findings indicate that first-trimester inflammatory indices, including CRP, did not show a significant association with CAPO occurrence.

When stratified by preterm birth status, inflammatory marker levels were significantly higher among patients who delivered before 37 weeks. Median NLR was significantly higher in the preterm group (4.84 Vs. 3.22; *p* = 0.0096). Median PLR (160.26 Vs. 129.17; *p* = 0.0037), MLR (0.39 Vs. 0.29; *p* = 0.0358), SII (1154.55 Vs. 784.08; *p* = 0.0044), and SIRI (2.91 Vs. 1.77; *p* = 0.0237) were also significantly elevated in preterm births (Table 2). Median CRP levels did not differ significantly between groups (2.43 Vs. 2.47; *p* = 0.4462). These findings suggest that first-trimester inflammatory indices, except for CRP, are significantly associated with preterm delivery among PREGDM pregnancies.

Notably, CRP levels did not show significant differences between CAPO-positive and negative groups or between preterm and term deliveries, underscoring its limited discriminative capacity in this cohort.

In the PREGDM group, preterm birth positively correlated with NLR (r = 0.22; *p* = 0.0091), PLR (r = 0.25; *p* = 0.0034), MLR (r = 0.18; *p* = 0.0352), SII (r = 0.24; *p* = 0.0041), and SIRI (r = 0.19; *p* = 0.0230). Negative correlations were observed between Apgar scores and NLR, PLR, SII, and SIRI. CRP showed no significant correlations with perinatal outcomes, except a borderline correlation with CAPO (r = 0.15; *p* = 0.081) (Table 3).

**Figure 1:**
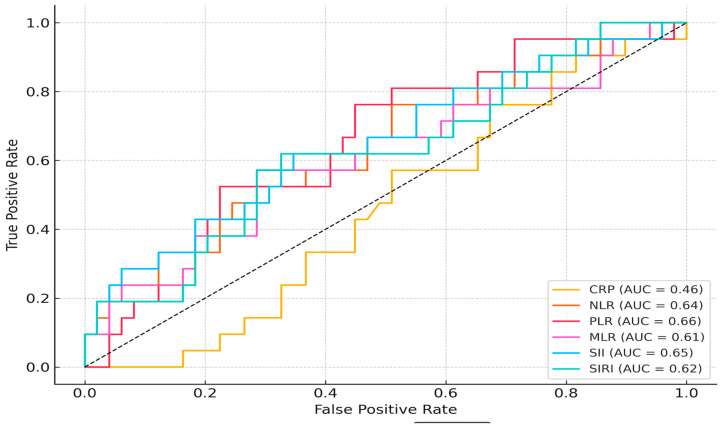
Receiver Operating Characteristic (ROC) Curves of Inflammatory Indices for Predicting Preterm Birth.

**Figure 2:**
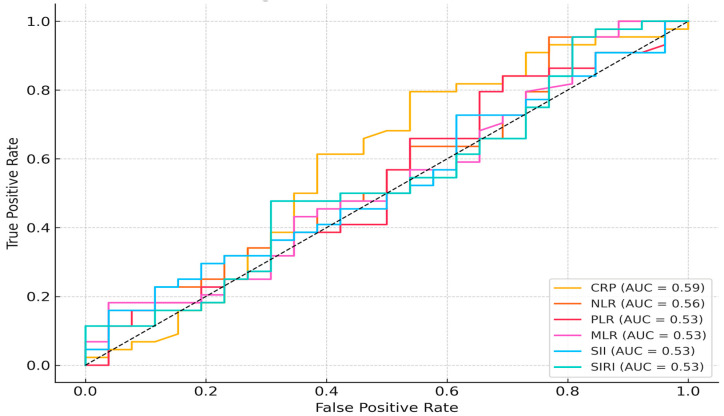
Receiver Operating Characteristic (ROC) Curves of Inflammatory Indices for Predicting Composite Adverse Perinatal Outcome (CAPO).

ROC analysis for preterm birth prediction showed moderate discriminative power for PLR (AUC = 0.655), SII (AUC = 0.652), and NLR (AUC = 0.638). MLR and SIRI had slightly lower AUC values (0.612 and 0.621, respectively), while CRP performed poorly (AUC = 0.459) (Figure 1). For CAPO prediction, all markers had limited predictive value, with AUCs ranging between 0.530 and 0.588 (Figure 2).

**Figure 3:**
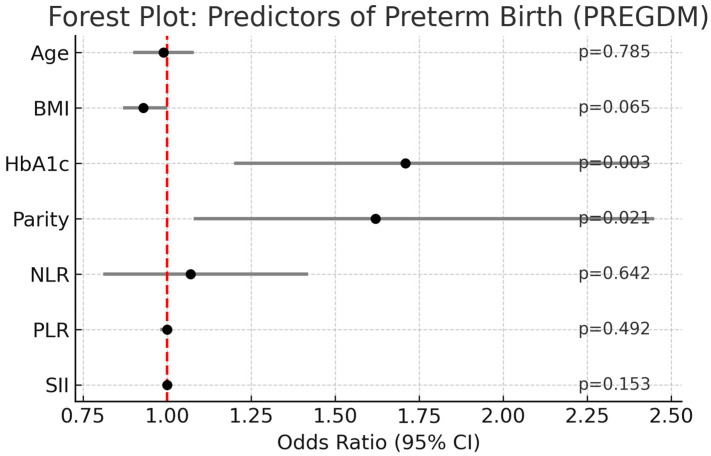
Forest plot of multivariate logistic regression analysis for predictors of preterm birth in pregnancies with PREGDM.

Multivariate logistic regression analysis was performed to adjust for potential confounders, including maternal age, BMI, HbA1c, and parity. In this model, NLR, PLR, and SII did not remain as independent predictors of preterm birth. Only HbA1c (OR: 1.71, 95% CI: 1.20–2.43, *p* = 0.003) and parity (OR: 1.62, 95% CI: 1.08–2.45, *p* = 0.021) were significant risk factors, while BMI showed a borderline association (*p* = 0.065) (Figure 3).

**Figure 4:**
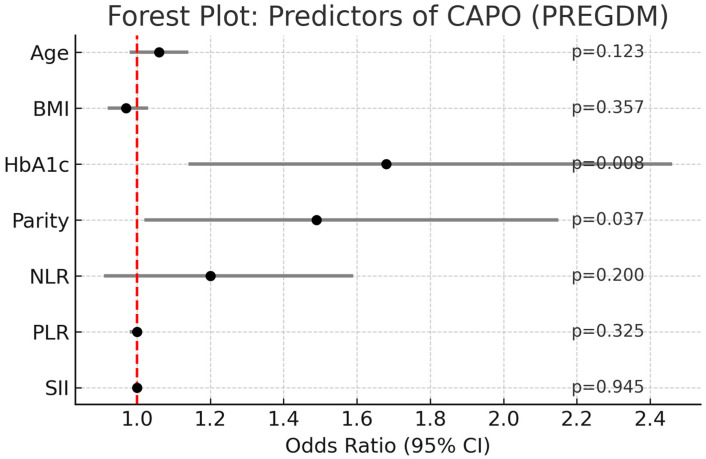
Forest plot of multivariate logistic regression analysis for predictors of CAPO in pregnancies with PREGDM.

Similarly, when CAPO was considered as the outcome, univariate analyses suggested higher inflammatory indices in adverse cases, but these associations were not retained after adjustment. In the multivariate model, only HbA1c (OR: 1.68, 95% CI: 1.14–2.46, *p* = 0.008) and parity (OR: 1.49, 95% CI: 1.02–2.15, *p* = 0.037) were identified as independent predictors of CAPO, whereas NLR, PLR, and SII were not statistically significant (Figure 4).

## 4. Discussion

This study demonstrated that early pregnancy inflammatory indices, including NLR, PLR, MLR, SII, and SIRI, were significantly higher in pregnancies complicated by PREGDM compared to healthy controls, reflecting a heightened systemic inflammatory state. These indices were also elevated in PREGDM patients who later experienced preterm delivery on univariate analysis. However, in multivariate analysis adjusted for maternal age, BMI, HbA1c, and parity, inflammatory indices did not remain independent predictors of preterm birth.

These findings suggest that chronic low-grade inflammation may contribute to the pathophysiology of preterm birth in PREGDM. Nevertheless, in our adjusted models, only HbA1c and parity were independent risk factors, while inflammatory indices lost statistical significance. This indicates that such markers reflect systemic inflammation but cannot serve as stand-alone predictors.

Previous studies have shown that increased NLR and PLR are associated with a higher risk of preterm labor in both diabetic and non-diabetic pregnancies [17,18]. In our cohort, however, these markers demonstrated only modest predictive performance for preterm birth, with AUC values ranging from 0.64 to 0.66. This indicates limited discriminative capacity rather than strong clinical utility. Other investigators have emphasized that combining sequential inflammatory markers throughout pregnancy may improve risk estimation [19]. In our study, we specifically focused on the early pregnancy period (8–14 weeks), a critical window for placental development and immune adaptation. While this approach allowed us to evaluate systemic inflammation at an earlier stage, the indices did not remain independent predictors after adjustment for confounders. These findings suggest that early inflammatory indices may be biologically relevant but insufficient on their own for reliable risk prediction, underscoring the need to integrate them into multiparametric models alongside established clinical and metabolic factors.

In contrast, inflammatory indices showed no predictive value for CAPO. This may be explained by the heterogeneity of outcomes within the composite endpoint. While macrosomia results from fetal hyperinsulinemia due to maternal hyperglycemia, intrauterine growth restriction is usually linked to placental insufficiency, and neonatal complications may have multifactorial origins. Such diverse mechanisms reduce the discriminative capacity of systemic inflammatory markers. Supporting this, other studies in PREGDM pregnancies have reported no consistent associations between inflammatory indices and composite neonatal outcomes, except for isolated findings such as an association between third-trimester NLR and neonatal respiratory compromise [17]. Similarly, prospective studies of early pregnancy NLR found no significant associations with small-for-gestational-age infants [20]. Taken together, these results emphasize that although biologically relevant, early hematologic indices have limited predictive accuracy for heterogeneous or multifactorial outcomes such as CAPO.

Numerous studies have examined these markers in hypertensive disorders and preeclampsia, where SII and PLR were higher in affected patients but did not remain independent predictors in multivariate analysis [21]. Other investigations observed associations between higher NLR or SII and fetal growth restriction, though their predictive value was limited when considered alone [22]. These results, consistent with ours, indicate that although inflammatory markers reflect underlying pathophysiology, they should not be used in isolation for clinical decision-making.

Early pregnancy SII and LMR have also been reported to be higher in women who subsequently developed gestational diabetes mellitus, suggesting that systemic inflammation may contribute to disturbances in glucose metabolism and adverse perinatal outcomes [23]. Our study, however, focused on pregestational diabetes and found that while inflammatory markers were associated with preterm birth in univariate analyses, they did not show independent predictive value once adjusted for key confounders.

Other research has shown that increased inflammatory indices may predict disease flares or neonatal complications in conditions such as inflammatory bowel disease [24] or preterm premature rupture of membranes [25], but again with limited accuracy when considered as single predictors. These findings, together with ours, suggest that individual blood-derived inflammatory indices have limited predictive accuracy when used alone, emphasizing the need for combining multiple markers or integrating them with established risk factors.

CRP did not differ significantly among outcome groups in our study, consistent with its known limitations as a non-specific acute-phase reactant influenced by transient maternal and environmental factors.

Overall, our findings demonstrate that early pregnancy hematologic indices may provide supplementary information in PREGDM pregnancies at increased risk of preterm delivery. However, only HbA1c and parity emerged as independent predictors, and inflammatory indices should be integrated into multiparametric models alongside clinical risk factors such as obstetric history, cervical length, glycemic control, and other biochemical or sonographic markers.

Our findings confirmed that HbA1c, rather than inflammatory indices, emerged as an independent predictor of preterm birth and CAPO in PREGDM pregnancies. This is consistent with recent evidence showing that inadequate glycemic control during early pregnancy substantially increases the risk of adverse outcomes. For instance, a large cohort study in type 1 diabetes demonstrated that higher first-trimester HbA1c was strongly associated with prematurity, LGA, and a composite criterion of complications, even when metabolic balance improved later in pregnancy [26]. Similarly, a recent investigation in mixed diabetic populations [27] reported that elevated HbA1c levels were predictive of preeclampsia, neonatal complications, and poor perinatal outcomes despite adjustment for confounders. Taken together, these data highlight that HbA1c reflects chronic glycemic exposure and is a more robust determinant of maternal–fetal complications than inflammatory indices. This may explain why, in our multivariate models, only HbA1c and parity retained independent predictive value, while systemic inflammatory markers lost significance.

Although parity was not significantly different between groups, it emerged as an independent predictor of preterm birth in the multivariate model. This discrepancy likely reflects confounding with other maternal characteristics such as age and metabolic status, which may have masked the effect of parity in crude comparisons. Adjustment for these covariates allowed the independent contribution of parity to be detected. Supporting this interpretation, large population-based studies have shown that both chronic and gestational diabetes across sequential pregnancies increase the recurrence risk of adverse outcomes including preterm birth and macrosomia, even when diabetes was present in only one pregnancy [28]. These findings suggest that reproductive history and interpregnancy metabolic exposures may modify perinatal risks, which could partially explain why parity remained significant after adjustment in our cohort.

The strengths of this study include its focus on early pregnancy measurements, enabling earlier risk stratification, and a robust comparison with a matched healthy control group. Limitations include its retrospective design, single-center nature, and potential residual confounding from unmeasured variables such as subclinical infections and nutritional status. The lack of association with CAPO underscores the need for refined outcome definitions and prospective validation. Another limitation of our study is that both type 1 and type 2 diabetic pregnancies were analyzed together under the category of PREGDM. However, it should be noted that the underlying pathophysiology differs substantially between these two groups. Type 1 diabetes is primarily characterized by autoimmune β-cell destruction and absolute insulin deficiency [29,30], whereas type 2 diabetes is typically associated with obesity, insulin resistance, and chronic low-grade inflammation [31]. These distinct mechanisms may influence systemic inflammatory responses in different ways and could partially explain the heterogeneity observed in our results. Future studies with larger sample sizes should consider stratified analyses of type 1 and type 2 diabetes to better clarify the specific contributions of systemic inflammation to adverse perinatal outcomes in each subgroup.

## 5. Conclusions

In conclusion, early pregnancy inflammatory indices such as NLR, PLR, and SII were associated with preterm birth in univariate analyses but did not remain independent predictors after adjustment for maternal and metabolic risk factors. HbA1c and parity emerged as the only independent predictors of adverse outcomes in pregnancies with PREGDM. Inflammatory indices were not predictive of CAPO, likely reflecting the heterogeneous pathophysiology of this composite measure. Overall, these findings indicate that hematologic indices primarily reflect systemic inflammation and may provide supplementary information, but they should not be used in isolation for clinical decision-making. Instead, they may be integrated into multiparametric models alongside established clinical and metabolic markers to improve risk assessment. Future large-scale, prospective, multicenter studies are warranted to validate these results and refine predictive models for early identification of high-risk pregnancies.

## Figures and Tables

**Table 1:** Comparison of demographic features, laboratory results, and complete blood cell indices between the study and control groups.

**Variable**	**PREGDM Mean ± SD/*n* (%)**	Control Mean ± SD/*n* (%)	***p*-Value**
Gravida	2.71 ± 1.67	1.66 ± 0.94	<0.0001
Parity	1.27 ± 1.25	0.53 ± 0.81	<0.0001
Abortus	0.44 ± 0.82	0.23 ± 0.66	0.0067
BMI (kg/m^2^)	30.64 ± 6.47	25.89 ± 1.97	<0.0001
HbA1c	6.56 ± 1.34	4.94 ± 0.36	<0.0001
FBG (mg/dL)	127.56 ± 51.84	82.56 ± 5.38	<0.0001
AST (IU/L)	16.01 ± 6.56	17.03 ± 6.69	0.1028
ALT (IU/L)	12.79 ± 6.55	16.51 ± 7.14	<0.0001
Hemoglobin (g/dL)	11.36 ± 1.35	11.52 ± 0.95	0.2482
WBC (/mm^3^)	11,710.94 ± 10,544.37	7598.41 ± 1640.81	<0.0001
Neutrophil (/mm^3^)	7155.14 ± 3198.07	4498.87 ± 928.09	<0.0001
Lymphocyte(/mm^3^)	1985.36 ± 1157.53	1843.50 ± 674.23	0.9653
Monocyte (/mm^3^)	657.36 ± 247.99	528.67 ± 140.72	<0.0001
Platelet (/mm^3^)	244,116.36 ± 89,056.15	215,628.46 ± 40,468.85	0.0135
CRP (mg/L)	2.53 ± 1.34	2.11 ± 1.37	0.0115
Gestational age at delivery (week)	36.69 ± 2.62	38.60 ± 0.95	<0.0001
Birth weight (gram)	3212.71 ± 747.90	3434.71 ± 341.24	0.0121
Apgar 1 min.	8.44 ± 1.47	8.96 ± 0.20	<0.0001
Apgar 5 min.	9.47 ± 1.44	9.93 ± 0.26	<0.0001
NLR	4.56 ± 3.51	2.68 ± 0.90	<0.0001
PLR	141.51 ± 54.12	128.13 ± 41.14	0.0213
MLR	0.40 ± 0.25	0.32 ± 0.13	0.0403
SII	1061.96 ± 809.16	573.19 ± 223.87	<0.0001
SIRI	3.18 ± 3.82	1.42 ± 0.63	<0.0001
Family History	68/140 (48.6%)	24/140 (17.1%)	<0.0001
Macrosomia	42/140 (30.0%)	6/140 (4.3%)	<0.0001
IUGR	6/140 (4.3%)	2/140 (1.4%)	0.2819
Cesarean birth	104/140 (74.3%)	62/140 (44.3%)	<0.0001
Preterm birth	42/140 (30.0%)	8/140 (5.7%)	<0.0001
NICU admission	30/140 (21.4%)	12/140 (8.6%)	0.0044
CAPO	88/140 (62.9%)	26/140 (18.6%)	<0.0001

Abbreviations: PREGDM, pregestational diabetes mellitus; BMI, body mass index; HbA1c, glycated hemoglobin; FBG, fasting blood glucose; AST, aspartate aminotransferase; ALT, alanine aminotransferase; WBC, white blood cell count; CRP, C-reactive protein; NLR, neutrophil-to-lymphocyte ratio; PLR, platelet-to-lymphocyte ratio; MLR, monocyte-to-lymphocyte ratio; SII, systemic immune–inflammation index; SIRI, systemic inflammation response index; IUGR, intrauterine growth restriction; NICU, neonatal intensive care unit; CAPO, composite adverse perinatal outcome.

**Table 2:** Comparison of Clinical and Inflammatory Markers in PREGDM Group (CAPO Positive vs. Negative and Preterm Positive vs. Negative.

**Variable**	**CAPO (+) Median**	**CAPO (−) Median**	CAPO *p*-Value	**Preterm (+) Median**	**Preterm (−) Median**	**Preterm *p*-Value**
Parity	1.00	1.00	0.0234	-	-	-
BMI (kg/m^2^)	-	-	-	26.40	31.00	0.002
HbA1c (%)	6.34	6.0	0.0393	6.60	6.10	0.009
FBG (mg/dl)	120.5	101.5	0.0012	142.00	102.00	<0.001
NLR	-	-	-	4.842	3.225	0.0096
PLR	-	-	-	160.265	129.167	0.0037
MLR	-	-	-	0.395	0.295	0.0358
SII	-	-	-	1154.551	784.080	0.0044
SIRI	-	-	-	2.907	1.767	0.0237

Abbreviations: BMI, body mass index; FBG, fasting blood glucose; NLR, neutrophil-to-lymphocyte ratio; PLR, platelet-to-lymphocyte ratio; MLR, monocyte-to-lymphocyte ratio; SII, systemic immune–inflammation index; SIRI, systemic inflammation response index; CAPO, composite adverse perinatal outcome. - Only statistically significant results are presented in the table.

**Table 3:** Significant Correlations Between Inflammatory Indices and Perinatal Outcomes in PREGDM Group.

Inflammatory Index	**Perinatal Outcome**	**Spearman r**	***p*-Value**
NLR	Preterm birth	0.22	0.0091
NLR	Apgar at 1 min	−0.22	0.0102
NLR	Apgar at 5 min	−0.20	0.0206
PLR	Preterm birth	0.25	0.0034
PLR	Apgar at 1 min	−0.22	0.0082
PLR	Apgar at 5 min	−0.25	0.0027
MLR	Preterm birth	0.18	0.0352
SII	Preterm birth	0.24	0.0041
SII	Apgar at 1 min	−0.21	0.0143
SII	Apgar at 5 min	−0.22	0.0076
SIRI	Preterm birth	0.19	0.0230
SIRI	Apgar at 1 min	−0.20	0.0171
SIRI	Apgar at 5 min	−0.19	0.0236

Abbreviations: NLR, neutrophil-to-lymphocyte ratio; PLR, platelet-to-lymphocyte ratio; MLR, monocyte-to-lymphocyte ratio; SII, systemic immune–inflammation index; SIRI, systemic inflammation response index.

## Data Availability

The datasets used and/or analyzed during the current study are available from the corresponding author upon reasonable request.

## Abbreviations

The following abbreviations are used in this manuscript:

PREGDM	Pregestational diabetes mellitus
NLR	Neutrophil-to-lymphocyte ratio
PLR	Platelet-to-lymphocyte ratio
MLR	Monocyte-to-lymphocyte ratio
SII	Systemic immune–inflammation index
SIRI	Systemic inflammation response index
CRP	C-reactive protein
APRI	AST to Platelet Ratio Index
CAPO	Composite adverse perinatal outcome
NICU	Neonatal intensive care unit
BMI	Body mass index
HbA1c	Glycated hemoglobin
RDS	Respiratory distress syndrome
AUC	Area under the curve
ROC	Receiver operating characteristic
SD	Standard deviation
